# The *Non-Coding RNA* Journal Club: Highlights on Recent Papers—14

**DOI:** 10.3390/ncrna11060075

**Published:** 2025-10-31

**Authors:** El Cheima Mhamedi, Florent Hubé, Suresh K. Alahari, Francisco J. Enguita, Barbara Pardini, Mark W. Feinberg, Laura Poliseno, Beshoy Armanios, Jing Jin, Xiao-Bo Zhong, Nikolaos Sideris, Salih Bayraktar, Leandro Castellano, Gaetano Santulli, Stanislovas S. Jankauskas, Will S. Plewa, Simon J. Conn, Ling Yang, Patrick K. T. Shiu, Abhishek Kaushik, Alexander Serganov, Massimo Gentile, Giuseppe Viglietto, Nicola Amodio, Tijana Mitić, Andrea Caporali

**Affiliations:** 1 Développement Adaptation et Vieillissement (Dev2a), CNRS UMR8263, INSERM U1345, IBPS, Sorbonne Université, 75005 Paris, France; elcheimamhamedi@gmail.com; 2 Department of Biochemistry and Molecular Biology, LSU School of Medicine, New Orleans, LA 70112, USA; 3 Faculdade de Medicina, Universidade de Lisboa, Av. Prof. Egas Moniz, 1649-028 Lisboa, Portugal; 4 Italian Institute for Genomic Medicine (IIGM), c/o IRCCS Candiolo, 10060 Turin, Italy; 5 Candiolo Cancer Institute, FPO IRCCS, Candiolo, 10060 Turin, Italy; 6 Brigham and Women’s Hospital, Division of Cardiovascular Medicine, Harvard Medical School, Boston, MA 02115, USA; 7 Institute of Clinical Physiology, CNR, 56124 Pisa, Italy; 8 Oncogenomics Unit, Core Research Laboratory, ISPRO, 56124 Pisa, Italy; 9 Department of Pharmaceutical Sciences, School of Pharmacy, The University of Connecticut, 69 N Eagliville Road, Storrs, CT 06269, USA; beshoy.armanios@uconn.edu (B.A.); jingjin.w@uconn.edu (J.J.); 10 School of Life Sciences, University of Sussex, Falmer, Brighton BN1 9QG, UKsb549@sussex.ac.uk (S.B.); 11 Albert Einstein College of Medicine, New York, NY 10461, USA; stanislovas.jankauskas@einsteinmed.edu; 12 Flinders Health and Medical Research Institute, College of Medicine & Public Health, Flinders University, Bedford Park, SA 5042, Australia; will.plewa@flinders.edu.au; 13 Department of Medical Genetics and Molecular Biochemistry, Lewis Katz School of Medicine at Temple University, Philadelphia, PA 19140, USA; 14 Division of Biological Sciences, University of Missouri, Columbia, MO 65211, USA; 15 Department of Biochemistry and Molecular Pharmacology, New York University Grossman School of Medicine, New York, NY 10016, USA; kaushik.bios@gmail.com; 16 Department of Pharmacy, Health and Nutritional Sciences, University of Calabria, 87036 Rende, Italy; massimo.gentile@unical.it; 17 Department of Experimental and Clinical Medicine, Magna Graecia University of Catanzaro, Viale Europa, Campus Germaneto, 88100 Catanzaro, Italy; viglietto@unicz.it; 18 Centre for Cardiovascular Science, Institute for Neuroscience and Cardiovascular Research, The University of Edinburgh, Edinburgh EH16 4TJ, UK; tijana.mitic@ed.ac.uk

## 1. Introduction

The field of non-coding RNA research is advancing at a breathtaking pace, continually uncovering new layers of regulatory complexity and functional diversity. Building upon the broad overview of recent progress presented in our previous Journal Club [1], the following highlights delve into specific, cutting-edge questions that are currently at the forefront of the field, and the papers reviewed herein exemplify the ongoing efforts to translate fundamental discoveries into a deeper mechanistic understanding and potential clinical applications.

## 2. Therapeutic Role of Pseudouridylation

### Highlight by El Cheima Mhamedi and Florent Hubé

Pseudouridylation (Ψ) is a structural RNA modification, emerging as a versatile player in gene translation regulation. Its absence can disrupt RNA biogenesis, leading to nonfunctional RNAs such as tRNA and rRNA. It also affects RNA stability, translatability, and pre-mRNA splicing. Beyond structural support, pseudouridylation is involved in fundamental cellular processes with pathological and therapeutic implications.

A particularly novel insight highlighted in Luo et al. [2] is the impact of pseudouridylation on stop codon decoding: targeted introduction of Ψ enables efficient readthrough, offering a new strategy to suppress nonsense mutations and premature termination codons. It thereby restores full-length protein expression allowing near-cognate tRNAs to recognize the modified stop codons.

Another key point is the role of Ψ in mitochondrial RNAs, which display a distinct modification profile compared to nuclear RNAs. The low abundance of Ψ in mitochondria is linked to the distribution and activity of Pseudouridine Synthases. Mutations in this enzyme impair protein synthesis, energy production and oxidative metabolism, impacting cell viability and contributing to diseases such as mitochondrial myopathy. All these lead to a unique Ψ landscape in mitochondria.

Finally, the article highlights advances in Ψ detection, such as direct RNA sequencing using Oxford Nanopore technology, which are essential for accurate and functional Ψ mapping. These discoveries confirm the central role of pseudouridylation in modern epitranscriptomics and its emerging therapeutic potential.

## 3. Relationship Between Target-Directed miRNA Degradation and Translation

### Highlight by Suresh K. Alahari

MicroRNAs (miRNAs) are small non-coding RNAs that play a pivotal role in controlling gene expression though binding to messenger RNAs. Argonaute proteins (AGOs) bind to miRNAs to form a complex. AGO-bound miRNAs interact with target messenger RNAs (mRNAs), leading to suppression of translation. Some short-lived miRNAs develop instability due to extensive base pairing with mRNAs, and this further leads to miRNA degradation, a process referred to as “target-directed miRNA degradation” (TDMD). Li and colleagues in a recent *Nature Communications* article reported that TDMD triggers degradation in the 3′untranslated region of miRNA to a much greater extent than that are in coding sequence (CDS) [3]. In this study, they have elegantly shown the relationship between translation and TDMD. Authors utilized accurate quantification by sequencing (AQ-seq), AGO cross-linking ligation and sequencing of miRNA-mRNA hybrids to derive the conclusion. Inhibition of translation enhanced TDMD efficacy by the CDS. Also, global blocking of translating ribosomes enhanced the efficacy of TDMD. Inhibiting global translation with inhibitors such as lactidomycin, harringtone and cycloheximide led CDS to trigger degradation of miR-16. Similar results were obtained for other TDMD-regulated miRNAs, miR-7 and miR-221. Furthermore, they explored other translation-sensitive miRNAs in mouse striatal cells and found that these were degraded by CDS. Based on this data, they conclude that very few miRNAs may be degraded by endogenous CDS triggers. Using Argonaute- Cross-linking, Ligation, and Sequencing of Hybrids (AGO-CLASH), the authors found that CDS triggers occur due to the binding of miR-17 family members (miR-17, miR-20a, miR-106 a) to TNFSF12 (tumor necrosis factor superfamily, member 12). In summary, this study reveals that naturally occurring CDS triggers are rare, but a very few examples can be found in AGO-CLASH hybrids.

## 4. Essential Long Non-Coding RNAs in Human Cells

### Highlight by Francisco J. Enguita

Long non-coding RNAs (lncRNAs) constitute a vast portion of the human transcriptome, yet their functional roles remain largely uncharacterized. Traditional DNA-targeting CRISPR approaches have provided limited insight, often confounded by local genomic effects. In this study, Liang et al. [4] employ transcriptome-scale RNA-targeting CRISPR-Cas13 screens across five human cell lines to systematically assess the contribution of over 6000 lncRNAs to cell fitness. The authors identified 778 essential lncRNAs, including 46 universally essential and over 700 that are cell-type-specific. Notably, most essential lncRNAs act independently of their neighboring protein-coding genes (PCGs), underscoring the specificity of RNA-level perturbation. Functional assays confirmed that lncRNA depletion impairs cell proliferation and survival, with effects on cell-cycle progression (G1 or G2/M arrest) and increased apoptosis. Single-cell transcriptomics (CaRPool-seq) revealed that lncRNA perturbation triggers transcriptional changes involving key regulatory pathways such as MYC, mTOR, p53, and cell-cycle checkpoints. Essential lncRNAs were found to be highly expressed during early development, co-expressed with proliferation-related genes, and dynamically regulated across tissues and stages. In cancer datasets from over 8800 primary tumors, many essential lncRNAs were differentially expressed and showed significant association with patient survival, supporting their potential as biomarkers and therapeutic targets [4]. This work represents the first comprehensive application of Cas13-based pooled screens for lncRNA function, offering high transcript specificity and avoiding artifacts linked to genomic proximity. It provides a rich catalog of essential human lncRNAs and demonstrates their roles in fundamental processes such as cell growth, development, and tumorigenesis, opening new avenues for non-coding RNA research and therapeutic innovation.

## 5. Circulating miRNA in Plasma: A New Hope for Non-Small Cell Lung Cancer?

### Highlight by Barbara Pardini

Early detection and accurate prediction of therapeutic responses is an urgent need for non-small cell lung cancer (NSCLC), one of the two most common types of lung cancer.

In light of improving patient diagnosis and outcomes, Abdipourbozorgbaghi and colleagues evaluated the potential of circulating microRNAs (miRNAs) in plasma from 78 NSCLC patients and 44 healthy controls for cancer diagnosis. The emerging panel of circulating miRNAs relevant for NSCLC has been further validated on 4000 NSCLC patients from an independent public database and miRNA expression levels have been correlated with clinicopathological information to identify independent prognostic miRNAs and those predictive of anti-PD-1 treatment response [5].

The authors demonstrated that circulating miRNAs can be used not only as novel biomarkers for the diagnosis of both lung adenocarcinoma (LUAD) and squamous cell carcinoma (LUSC) but also for treatment management of NSCLC. In fact, miR-135b-5p, miR-196a-5p, miR-31-5p (LUAD), and miR-205 (LUSC) have been shown to serve as independent prognostic markers for survival and two miRNA clusters (miR-183/96/182 and miR-767/105) exhibit predictive potential in anti-PD-1-treated LUAD patients.

Circulating miRNAs have enormous potential as biomarkers, and the work of Abdipourbozorgbaghi and colleagues demonstrated their importance in the management of NSCLC, from diagnosis to treatment and monitoring.

Circulating miRNA profiles specifically tailored for NSCLC diagnosis have been shown to have high sensitivity and specificity and can be used as both independent prognostic biomarkers and predictors of response to immune-checkpoint inhibitors.

## 6. Turning on Non-Coding RNA Loci for Therapeutic Intervention

### Highlight by Mark W. Feinberg

Accumulating studies highlight a growing list of multi-transcript loci comprising both long non-coding RNAs (lncRNAs) and microRNAs (miRNAs) within the same genomic location [6,7,8,9]. Similar to protein-coding transcripts, these regions of multi-component non-coding RNAs (ncRNAs) are regulated by cis-acting regulating elements (CREs) typically found upstream to the locus and can lead to a broad transcriptional response across the entire ncRNA locus. For example, the *MIR503HG* lncRNA locus harbors two miRNAs, *miR-424* and *miR-503*, that are evolutionarily conserved. Loss of *MIR503HG* has been implicated in cellular responses involved in tumorigenesis, adverse vascular remodeling, and left ventricular malformation during cardiac development [6,10,11]. Interestingly, overexpression of *MIR503HG* or separately *miR-424/miR-503* have been shown to regulate different aspects in the pathobiology of a mouse model of pulmonary artery hypertension, raising the possibility that both may be needed for maximal therapeutic impact [6,12]. How then can such a genomic ncRNA locus be “turned-on” for therapeutic gain?

In the journal *Vascular Pharmacology*, Monteiro et al. [13] present a novel CRISPR activation (CRISPRa)-based strategy termed “cis-ON” to target and transcriptionally activate the entire *MIR503HG* locus containing the lncRNA *MIR503HG* and the two embedded microRNAs, *miR-424* and *miR-503*. After identifying the major promoter region of *MIR403HG* through integrative epigenomic analysis, the authors used a streamlined, single-vector lentiviral system co-expressing a dead Cas9 (dCas9)-VP64-p65-Rta(VPR), lacking its endonuclease activity, and a single guide RNA (sgRNA) to target a shared CRE and validated the system in primary endothelial cells. The platform achieved robust transcriptional upregulation of *MIR503HG* and the two embedded miRNAs, *miR-424* and *miR-503*, while sparing the neighboring *LINC00629* lncRNA locus, demonstrating specificity. This methodological report highlights the feasibility of activating a multi-transcript ncRNA loci through a single CRE, bypassing the need to manipulate each transcript individually. However, some limitations should be noted: functional and phenotypic outcomes of the locus-wide activation were not explored, the long-term stability and epigenetic effects of this CRISPRa (cis-ON) platform remain unclear, broader specificity was not shown for potentially shared CREs, and how to “turn-off” such an approach will require further study. Activating specific lncRNA isoforms may also be challenging. Finally, careful selection of activating ncRNA loci will be important since there are examples of the lncRNA and the miRNA having antithetical effects on cellular processes (e.g., *MIR181A1HG* and *miR-181a/b* in vascular inflammation) [9,14,15,16,17,18].

Collectively, this work establishes a foundation for therapeutically “turning-on” a complex ncRNA locus. Future studies will be of interest on how this can be translated to “fine-tune” transcriptional responses for precision control in a variety of disease states.

## 7. Aptamers and Bugs Team up for Drug Delivery in Pancreatic Cancer

### Highlight by Laura Poliseno

In their article “Aptamer-drug conjugates-loaded bacteria for pancreatic cancer synergistic therapy” [19], Xiao and colleagues devise an elegant strategy to ensure the selective delivery of MMAE (an inhibitor of tubulin polymerization) to the pancreatic cancer microenvironment. The drug is conjugated to Sgc8c, an aptamer that binds to the PTK7 protein on the surface of pancreatic cancer cells. Then, a bio-orthogonal and bio-compatible click-chemistry reaction is used to covalently load the Sgc8c-MMAE aptamer-drug conjugate on the surface of VNP20009 (VNP for short) [20]. VNP is an attenuated strain of *Salmonella typhimurium*. It preferentially accumulates in stiff and hypoxic cancer microenvironments, where it kills cancer cells through both cell-autonomous mechanisms (inducing apoptosis and necrosis) and non-cell-autonomous mechanisms (triggering the host immune response against infected cancer cells).

The loaded bacterium (VNP@Sgc8c-MMAE) was tested in several mouse models of pancreatic cancer. Injected intravenously into the therapeutic setting, it exhibits negligible toxicity and much stronger anticancer activity compared to both VNP and Sgc8c-MMAE. On one side, the aptamer promotes the accumulation of the bacterium and the drug in the cancer microenvironment, thanks to the recognition of the PTK7 surface protein. On the other hand, the loading on the VNP surface is favorable for both the aptamer and the drug: the aptamer becomes more stable and persists much longer after the injection, while the drug can reach the inner layers of the tumor mass. In addition, the host immune system is awakened and reacts against the VNP-infected cancer cells.

In conclusion, this article shows the potential of bugs and aptamers as “intelligent carriers” for the selective delivery of the drug of choice to the cancer microenvironment. It also shows that this approach integrates chemotherapy with immunotherapy, resulting in enhanced anticancer activity even in hard-to-treat cancer types like pancreatic cancer.

## 8. Functional Conservation of lncRNAs Between Human and Mouse in the Regulation of Metabolic Functions in the Liver

### Highlight by Beshoy Armanios, Jing Jin, and Xiao-bo Zhong

A key challenge in long non-coding RNA (lncRNA) biology is the lack of sequence conservation across species, which limits the ability to investigate human lncRNA functions in mouse models. In a recent study published in *Gastroenterology* [21], Dr. Haiming Cao’s team addressed this fundamental challenge. Leveraging a sequence-independent and function-based strategy, the team identifies a novel class of functionally conserved lncRNA metabolic regulators (fcLMRs) that exhibit conserved metabolic roles in human and mouse livers, even though the lncRNAs show little or no primary sequence similarity.

By integrating transcriptomic data from humanized mice, RNA-seq, and gene network analyses, the study systematically identifies syntenic lncRNA pairs with similar metabolic regulatory patterns. Of these, the human/mouse LMR1 pair emerged as a key regulator of lipid metabolism. Functional studies demonstrated that both human and mouse LMR1 lncRNAs suppress protein translation by binding the poly(A)-binding protein cytoplasmic 1 (PABPC1), mediated by conserved short RNA structural motifs. This repression elevates intracellular amino acid levels, thereby activating the mammalian target of rapamycin–sterol regulatory element binding protein 1 (mTOR–SREBP1) axis, a key driver of lipogenesis. Rescue experiments confirm that the human lncRNA can substitute for its murine counterpart in vivo, offering a translationally relevant model for preclinical studies.

Strikingly, the authors show that these lncRNAs are upregulated in hepatic steatosis models and in patients with metabolic dysfunctional fatty liver disease (MAFLD), positioning hLMR1 as a potential therapeutic target. The findings highlight an unappreciated layer of regulatory conservation rooted in RNA structures and functions rather than sequences, and they offer a roadmap for discovering and validating non-coding RNA drug targets using cross-species models.

By revealing that lncRNA functions can be conserved at the level of RNA structures and motif-based interactions, not just sequences, this work lays the foundation for identifying human disease-relevant lncRNAs and validating their roles, using well-established mouse models.

## 9. miR-9 Moves In: A MicroRNA Remodels the Genome from Within

### Highlight by Nikolaos Sideris, Salih Bayraktar and Leandro Castellano

In a compelling advance, Cordero and colleagues reveal a nuclear role for mature microRNA-9 (miR-9) in orchestrating three-dimensional chromatin architecture to drive gene activation in response to TGF-β1 in human lung fibroblasts (hLFs) [22]. Traditionally viewed as a cytoplasmic post-transcriptional regulator, miR-9 is shown here to translocate into the nucleus in idiopathic pulmonary fibrosis (IPF) cells, where it binds both promoters and super-enhancers of TGF-β-responsive genes. Using chromatin isolation by RNA purification sequencing (ChIRP-seq), the authors demonstrate that miR-9 associates with DNA regions enriched in G-quadruplex (G4) structures, particularly at loci upregulated by TGF-β1. These G4-positive regions also exhibit strong enrichment for euchromatic and super-enhancer markers, including H3K4me3, H3K27ac, and MED1, as shown by G4 ChIP-seq and CUT&Tag profiling. Crucially, nuclear miR-9 is required for G4 formation or stabilization at its chromatin binding sites. Loss of miR-9 leads to reduced G4 signal, decreased H3K27ac enrichment, and impaired recruitment of RNA Polymerase II to target genes. These changes disrupt enhancer-promoter interactions and block transcriptional activation. Conversely, TGF-β1 stimulation increases nuclear levels of mature miR-9 and enhances its chromatin association at target loci. This is accompanied by elevated G4 formation and increased enrichment of active histone marks, as confirmed by G4 ChIP-seq and CUT&Tag. The authors further show that miR-9-bound regions correspond to TGF-β1 induced genes. Chromatin conformation capture assays confirm that these chromatin interactions depend on miR-9 and the presence of G4s, and disruption of this interaction abolishes chromatin contacts and transcriptional upregulation. These findings provide a mechanistic framework for how TGF-β1 may drive transcriptional reprogramming in hyperproliferative disorders such as IPF or cancer, where nuclear accumulation of miR-9 is observed. Furthermore, this study also establishes nuclear miR-9 as a structural RNA that facilitates spatial genome reorganization and transcriptional control, expanding the known functional repertoire of microRNAs in mammalian cells.

## 10. Muscles Sabotage Their Own Blood Supply via Extracellular Vesicles Enriched in miR-499-5p: Signed, Sealed, and Returned to Sender

### Highlight by Gaetano Santulli and Stanislovas Jankauskas

Critical limb ischemia (CLI) remains a major source of morbidity and mortality in patients with diabetes mellitus, with poor therapeutic outcomes largely due to impaired neovascularization in ischemic tissues. In a recent study [23], Cheng and collaborators provide compelling mechanistic insight into this pathophysiology by demonstrating that skeletal muscle cells (SKMCs) from diabetic mice secrete small extracellular vesicles (sEVs) enriched with miR-499-5p, which are taken up by endothelial cells (ECs), thereby suppressing angiogenesis. Using the db/db mouse model of type 2 diabetes and surgically induced hindlimb ischemia, the authors determined that diabetic SKMCs overexpress miR-499-5p and that ischemia further elevates this miR in both SKMCs and ECs. Functionally, the overexpression of miR-499-5p leads to diminished EC angiogenic capacity, impaired tube formation, and defective perfusion recovery. Mechanistically, they identified SOX6 as a direct miR-499-5p target; restoring SOX6 levels or silencing miR-499-5p rescued angiogenic function in vitro and in vivo. These findings suggest that pathological SKMC-to-EC communication via sEV-packaged miR-499-5p represents a previously unrecognized contributor to the vascular complications of diabetes.

This study is notable for providing a muscle-centric mechanism of vascular dysfunction, underscoring the importance of intercellular signaling in diabetic tissue injury. It also broadens the scope of therapeutic targeting by suggesting that local inhibition of miR-499-5p, blockade of sEV release, or SOX6 restoration may constitute promising strategies to restore neovascularization in diabetic CLI. The observation that muscle-specific miR-499-5p can act remotely on ECs challenges traditional assumptions about tissue-specific miRs and highlights sEVs as critical mediators of tissue cross-talk. Overall, this study advances our understanding of diabetic vascular disease and highlights novel muscle–endothelium communication pathways that may serve as therapeutic targets for CLI in diabetes.

## 11. Circular RNAs Are Definitely Not Nonsense

### Highlight by Will S. Plewa and Simon J. Conn.

The breadth of the circular RNA (circRNA) interactome has grown significantly over the past decade to include thousands of RNA, protein, and DNA targets [24]. With circRNAs long believed to be accidents of mis-splicing that could generate new splicing isoforms of the cognate RNA transcript, it remained unanswered what the in-terplay was between circRNAs and nonsense-mediated decay (NMD). In a recent study published in *Molecular Cell*, Boo et al. have revealed a novel role for endogenous circRNAs in triggering NMD, not for the parent transcript, but for target mRNAs bound by the circRNA within their 3′ untranslated regions [25]. This process, called circNMD, arises from the formation of circRNA:RNA duplexes, with up to 3 mis-matches within a 21 nucleotide stretch. This sequence-specific post-transcriptional regulatory mechanism by circRNAs on cell homeostasis is an additional function to consider in understanding their biological relevance.

The study identifies two circRNAs that interact with BCL2L11, a pro-apoptotic mRNA, elegantly demonstrating that exogenous circRNAs can influence cellular apoptosis via the circNMD pathway. Exploiting this phenomenon, the authors engi-neered several circRNAs that were able to downregulate target endogenous mRNAs.

These findings further expand the functional purview of circRNAs and open the door for review of their functions well beyond their ostensible role as micro-RNA sponges. By uncovering a fundamental function of circRNAs in mRNA quality control surveillance, this work stands as a promising proof-of-concept for circRNA-based therapeutics.

## 12. A New Approach to Identify Functionally Conserved lncRNA

### Highlight by Ling Yang

Identifying functionally—but not sequence—conserved long non-coding RNAs (lncRNAs) remains a major challenge. Encouragingly, a recent study by Jiang et al., published in *Gastroenterology*, presents a practical strategy to systematically identify functionally conserved lncRNA metabolic regulators (fcLMRs) in the liver [21].

In this study, the authors employed a liver humanized mouse model—containing human hepatocytes within the mouse liver—to identify syntenic human and mouse lncRNAs that respond to metabolic stimuli in parallel and are associated with the same metabolic pathways. Through this approach, they identified 59 potential fcLMRs. Four of these candidates underwent experimental validation, and three were confirmed as functionally conserved using a rescue-based strategy.

Mechanistically, the authors demonstrated that one validated fcLMR pair exerts its function by interacting with the same protein, PABPC1, to regulate hepatic lipid metabolism. Furthermore, the fcLMR-PABPC1 complex was found to activate the mTOR-SREBP1 axis to regulate lipogenic gene expression.

Taken together, this study sheds light on the functional study of human lncRNAs without mouse homologs under metabolic conditions. The identification of fcLMR pairs in the liver could accelerate our understanding of lncRNA’s roles in liver physiology and aid in the development of new therapeutic strategies for liver diseases.

## 13. A Potential Adjuvant for RNA Silencing Therapies

### Highlight by Patrick K. T. Shiu

Since the discovery of microRNAs (miRNAs), very few factors have been shown to inhibit their accumulation in a broad manner. In a recent study published in *Science*, Seungjae Lee and others uncovered an unexpected repressor of the miRNA pathway that could be exploited for small RNA therapies [26].

5-aminolevulinic acid synthase 1 (ALAS1) is the initiating enzyme for the biosynthetic pathway of heme, which is a cofactor for the Microprocessor complex (the machinery for cleaving primary miRNAs into precursor miRNAs). Surprisingly, human *ALAS1-KO* (knockout) cells cultured in heme-replete media have elevated levels of mature miRNAs. Knockout of another heme biosynthetic enzyme does not have the same effect, suggesting that ALAS1 hinders miRNA production with a secondary function (outside of heme biosynthesis). The loss of ALAS1 enhances the assembly of the RNA-induced silencing complex (RISC; which turns a Dicer-cut miRNA duplex into an active single-stranded RNA guide), providing an explanation for the increased abundance of mature miRNAs. Interestingly, while mature ALAS1 mediates heme biosynthesis inside mitochondria, the cytoplasmic ALAS1 precursor appears to down-regulate the protein level of Argonaute 2 (a key component of RISC).

The findings here suggest that ALAS1 depletion may possibly enhance RNA interference (RNAi)-based therapeutics. Indeed, the addition of synthetic *Alas1/2*-siRNAs (small interfering RNAs), which knock down the total cellular amount of ALAS, can boost the efficacy of another siRNA compound in mice. Coincidentally, an *ALAS1*-siRNA drug called givosiran is already FDA-approved (to treat acute hepatic porphyrias). Future investigation into the use of givosiran as an adjuvant for siRNA drugs could improve their effectiveness and open up new target organs for RNAi therapies.

## 14. Cryo-EM Reveals High-Order Assemblies of Natural RNAs

### Highlighted by Abhishek Kaushik and Alexander Serganov

As bacteria and phages extensively use various non-coding RNAs for gene expression control and many other cellular activities, every novel RNA attracts significant interest from the research community to understand its biological role. Among these RNAs are large conserved RNAs coined GOLLD (Giant, Ornate, Lake- and Lactobacillales-Derived), ROOL (Rumen-Originating, Ornate, Large), OLE (Ornate Large Extremophilic), and ARRPOF (area required for replication in a plasmid of *Fusobacterium*), previously identified through covariance analysis of genomic and metagenomic sequences [27,28,29]. These results and follow-up experiments highlighted extensive secondary structures of these RNAs and hinted at their involvement in cellular functions, but their precise biological roles remain to be understood. Three recent studies [30,31,32] used cryo-electron microscopy (cryo-EM) to obtain the first insights into the structural organization of these molecules. The researchers uncovered that these RNAs assemble into intricate, homo-oligomeric structures in the absence of associated proteins—a novel observation in natural RNA molecules. Cryo-EM structures revealed distinctive architectures: for example, OLE could form a dimeric “bundle of pipes”, interconnected at the ends via a five-way junction; ROOL assembles into larger, hollow hexameric or octameric nanocages; GOLLD forms dodecameric or 14-mer nanocages, featuring internal hollows with some disordered loops, and ARRPOF forms a ring-like homodimer. These symmetric quaternary assemblies are stabilized by many tertiary and quaternary intermolecular contacts, offering a rich source of data for RNA structure prediction and design efforts. Interestingly, the assemblies could differ in complexity, size, and overall conformation in solution. Together, these works will enable future research aimed at elucidating the biological function of these RNA molecules and spearhead the engineering of RNA nanocages as delivery vesicles for academic research and therapeutic applications.

## 15. The Hidden Code: Long Non-Coding RNA Isoform Vulnerabilities in Multiple Myeloma

### Highlight by Massimo Gentile, Giuseppe Viglietto and Nicola Amodio

Long non-coding RNAs (lncRNAs) are pleiotropic regulators that comprise a substantial portion of the human transcriptome, yet their precise roles in pathophysiological processes remain largely undefined. In a recent study published in *Blood*, Morelli et al. leveraged an RNA-targeting CRISPR-Cas13d system to systematically identify hundreds of tumor-essential (te-) lncRNA isoforms exhibiting potential clinical relevance [33].

The developed technology, which overcomes the limitations of DNA-targeting approaches such as CRISPR-Cas9 and CRISPR interference (CRISPRi), enabled the authors to achieve, for the first time, isoform-level identification of lncRNAs. Among the te-lncRNAs uncovered, the authors distinguished between pan-cancer and multiple myeloma (MM)–specific isoforms. These were further characterized for their prognostic significance through analyses of the IFM-DFCI2009 and CoMMpass clinical datasets.

By employing isoform-specific targeting approaches, the authors functionally validated SNHG6-003—a cancer-associated isoform of the lncRNA SNHG6—as a critical dependency across MM cell lines. Subcellular RNA sequencing revealed high expression levels of SNHG6-003 and its preferential localization to the endoplasmic reticulum (ER), where it engages with heat shock proteins to regulate proteostasis. To dissect its cytoplasmic functions, a CRISPR-Cas13d system modified with a nuclear export signal was utilized, further substantiating the reliance of MM cells on SNHG6-003 for proliferation. In clinical samples, SNHG6-003 was significantly upregulated in plasma cells from both newly diagnosed and relapsed/refractory MM patients. Targeted silencing of SNHG6-003 using LNA gapmer antisense oligonucleotides (ASOs) induced potent anti-MM effects, circumventing the protective role of the tumor microenvironment.

Collectively, these findings underscore the pivotal role of isoform-specific regulation in determining lncRNA function and open new avenues for the identification of therapeutically actionable dependencies in cancer. To facilitate further research, the authors have developed the LongDEP Portal, an open-access platform that offers comprehensive datasets on the expression patterns, clinical correlations, subcellular distribution, and functional screening of te-lncRNAs.

## 16. Decoding the Regulatory Potential of Enhancer-Associated Long Non-Coding RNAs in Vascular Remodeling

### Highlight by Tijana Mitić and Andrea Caporali

Extensive research has demonstrated that long non-coding RNAs (lncRNAs) play an essential role in genome organization, cell structure, and modulation of gene expression. They directly interact with DNA, RNA, and/or proteins, often involving repetitive elements. Enhancer-associated lncRNAs (elncRNAs) are transcribed from active enhancer regions and are particularly important for gene regulation, as they stabilize enhancer-promoter looping, affect chromatin accessibility, and recruit transcription factors.

The study of Guo et al. identifies LncRNA-ITGA2 as a novel elncRNA that is highly upregulated in proliferative vascular smooth muscle cells (VSMCs) and coronary arteries of patients with coronary artery disease (CAD) [34]. Mechanistically, LncRNA-ITGA2 facilitates enhancer-promoter interactions at the *ITGA2* gene locus in a manner dependent on the DNA-binding protein, NONO (non-pou domain containing octamer-binding protein). This increases ITGA2 transcription and activates the ITGA2/FAK/ERK signaling pathway, promoting VSMC proliferation and migration–key processes in vascular remodeling, such as atherosclerosis and restenosis. Both in vitro and in vivo approaches demonstrate that overexpression of LncRNA-ITGA2 increases VSMC proliferation, migration, and neointimal hyperplasia. Conversely, knockdown or knockout of LncRNA-ITGA2, or disruption of its regulatory components, suppresses these pathogenic processes. Notably, the regulatory mechanism of LncRNA-ITGA2 is conserved across species and exhibits clinical relevance in human cardiovascular disease. The findings imply LncRNA-ITGA2 could serve as a promising diagnostic and therapeutic target for vascular diseases by disrupting the abnormal VSMC growth and migration pathways that underlie conditions such as atherosclerosis and restenosis.

Further in-depth mechanistic investigations and translational research could establish LncRNA-ITGA2 as a promising new frontier in vascular medicine. This could potentially influence treatment approaches for atherosclerosis and restenosis. Specifically, it would be interesting to develop targeted therapeutic strategies, using antisense oligonucleotides or RNA-targeted gene therapies, to inhibit the activity of LncRNA-ITGA2 and reduce neointimal hyperplasia or prevent restenosis.
